# Australian Maltreated Infants and Young Children Can Achieve Positive Relational Health With Neurodevelopmentally- and Trauma-Informed Interventions Provided Within Relationally-Positive and Stable Environments

**DOI:** 10.3389/fpsyt.2021.680343

**Published:** 2021-07-28

**Authors:** Allison Cox, Margarita Frederico, Holly Mosse, Lyn Radford, Dallas Ambry, Clare Ryan

**Affiliations:** ^1^Berry Street Take Two Program, Eaglemont, VIC, Australia; ^2^Department of Occupational Therapy and Social Work and Social Policy, School of Allied Health, Human Services and Sport, College of Science, Health and Engineering LaTrobe University, Bundoora, VIC, Australia

**Keywords:** maltreatment, developmental trauma, relational health, neurosequential model of therapeutics, trauma informed approaches

## Abstract

**Background:** Childhood maltreatment such as abuse, neglect and family violence has a profound impact on children's psychological and relational functioning and their lifelong trajectory, with associated adverse physical and mental health outcomes, higher mortality rates and reduced socioeconomic opportunities. The aim of the study was to explore the impact of neurodevelopmentally- and trauma-informed interventions on the relational health of children who have experienced maltreatment.

**Context:** The study was conducted at Berry Street Take Two, an Australian therapeutic service. Take Two provides services to Victorian children aged 0-18 years, to address the impact of the trauma they have experienced from maltreatment. Take Two clinicians use relational and ecological frameworks, neurodevelopmental research and evidence-informed approaches to repair family relationships and develop networks of caring adults that focus on meeting the child's needs. Take Two uses the NMT approach as a framework for clinical intervention-planning and is site-certified in the use of the NMT Clinical Practice tools.

**Method:** The mixed methods study had two components. A cross sectional study of baseline and repeat clinical measure data (HoNOSCA and SDQ) with a cohort of children aged 2–11 years (*n* = 91), who were clients of Berry Street Take Two between 2014 and 2019, was conducted utilizing SPSS. The quantitative data analysis was supplemented by three case studies of Berry Street Take Two clients, which explored the process of intervention, including intervention type, timing and dosage. The case studies drew on the full case record for each child to illustrate the impact of NMT-informed interventions on the relational health, psychological and behavioral functioning of children.

**Results:** The study found that Take Two intervention was associated with improved relational health, measured by the NMT metric and supported by significant positive changes on the SDQ and HoNOSCA with medium effect sizes (cohen's *d*). The case study analysis highlighted the importance of intervention addressing individual, family and systems elements to bring about positive change.

**Conclusions:** This study illustrates the value of neurodevelopmental trauma-informed interventions in positively impacting on the relational health and current functioning of maltreated children and the potential to reduce the lifelong impact of maltreatment.

## Introduction

Childhood maltreatment is a complex and challenging issue worldwide. There is a link between childhood maltreatment and adverse life outcomes, with a corresponding high cost to society, both in terms of economics and human suffering (1). Maltreatment, such as abuse, neglect and/or family violence, profoundly impacts on psychological, developmental and relational health (2). This impact on children's developmental trajectories frequently carries over into adolescence and adulthood, where issues can be compounded and entrenched into lifelong adverse outcomes (1). Hillis et al. (3) estimated that over one billion children globally were currently experiencing violence and abuse. Moreover, the cost to communities of the consequences of child maltreatment is high. The annual estimated cost to the USA community was $2 trillion USD (4) and $7.7 billion AUD in Australia (5).

The complexity of the issues related to childhood maltreatment, combined with the heterogeneity of maltreatment experiences and the complexity of their intersection with, and impact on, developmental trajectories, makes researching this area a formidable task (6). Despite this complexity there are several paths of enquiry in the literature which show promising evidence for interventions to mitigate and ameliorate the impacts of childhood maltreatment. One of these areas for enquiry is that of relational health, and how positive relational experiences, provided within a safe and stable environment, can help children to overcome the impacts of maltreatment experiences (6).

The aim of the current study was to explore the impact of individually tailored neurodevelopmental and trauma-informed relational interventions on a cohort of Australian children who have experienced maltreatment.

## Literature Review

### Prevalence of Maltreatment in Australia

Tanaka et al. (7) noted that the prevalence of childhood maltreatment is underestimated. Challenges in ascertaining the extent of childhood maltreatment include issues of definitions, cultural norms, reporting rates, data sources, and challenges in assessing severity and frequency of maltreatment experiences (8). Research has highlighted that data captured from Child Protective services does not correlate with self-report data (9). Despite these challenges, the data that is available demonstrates that childhood maltreatment is sadly a pervasive and all too common experience for many children.

The Australian Institute of Family Studies (8) conducted an analysis of published research which showed promise in determining the rates of five types of maltreatment; physical; sexual; emotional; family violence exposure and neglect. The exposure of Australian children to family violence is estimated to be between 4 and 23% with variability in study design accounting for the wide variance. It is estimated that between 5 and 10% of Australian children aged 0–18 years are physically abused (8), with abuse estimates ranging from 9 to 14%. The prevalence of child sexual abuse is variable depending on gender and other factors but is estimated to range between 1 and 26% of the Australian population. The least reported type of maltreatment was neglect, with a prevalence rate of 1.6–4.0%. While the results from this study can only be described as suggestive as opposed to conclusive, they give a glimpse into the prevalence of child maltreatment in the Australian context.

Determining the prevalence rates of sexual abuse of Australian children is complicated by contested definitions and under-reporting. When broad definitions of abuse and neglect are applied prevalence rates can be up to 45% for females (8). When more specific definitions are used it was found that prevalence rates for males were in the range of 1.4–7.5% for abuse involving penetration and in the range of 5.2–12% for non-penetrative abuse, with female rates being 4.0–12.0 and 14–26.8%, respectively (8). An Australian Bureau of Statistics survey conducted with 17,050 adults asking about their childhood experience of maltreatment indicated 13% of the Australian adult population had experienced physical and/or sexual abuse as children (8).

In the 2017–2018 year ~2.87% (159,000 children) of Australian children were subject to Child Protection interventions such as investigations and care/protective orders. Children who identify as Aboriginal and/or Torres Strait Islander were eight times more likely to be involved in Child Protection Services (10). This reflects the ongoing impact of invasion and forced colonization and past genocidal policies of the forced removal of children, known in Australia as the Stolen Generations (11). Children from regional and remote areas are also over-represented in child protection and out of home care services (8).

### Impact of Maltreatment

Child maltreatment can have a pervasive neurophysiological impact on the developing child (2, 12). Children who experience complex trauma may experience developmental disruption and difficulties across “domains of impairment” [(13), p. 392] including: attachment, affect regulation, dissociation, behavioral control, cognition, and self-concept. While the precise pathways through which complex trauma produces psychopathology are yet to be elucidated, it is hypothesized that interactions between individual genetic differences and environmental stressors influence the neurobiological mechanisms that underlie psychological and emotional development (14).

Subsequently, children who have suffered maltreatment have a significantly higher risk than the general population of developing physical and mental health difficulties, such as mood, personality and anxiety disorders, schizophrenia, and post-traumatic stress disorder (5, 15–21). Another large cohort study comparing 2,759 sexual abuse victims with control subjects found that the experience of sexual abuse continued to be associated with psychosis, affective and anxiety disorders, substance abuse and personality disorders through adolescence and into adulthood (22).

Additional to the neurobiological and psychological impacts, maltreated children are also more likely to have compromised social functioning due to impeded coping strategies and behavioral self-control (23, 24). As a consequence, their relationships with others, including caregivers, can be compromised. This includes “altered help-seeking” behavior where children who suffer maltreatment become excessively dependent upon others or, conversely, develop antisocial behaviors, including violence, leading to rejection and social isolation (13, 25–27). A recent Australian study (28) demonstrated that children who were involved with Child Protection services were significantly more likely to have subsequent involvement with justice and homelessness services. Additionally, these children also had lower literacy and numeracy levels than the general population, leading to relatively poor educational and economic outcomes throughout life (29), further exacerbating their adverse outcomes.

Maltreated children also frequently experience adverse impacts of family separation and negative experiences of the child protection system. These additional stressors, added to the already substantial trauma from the abuse and neglect, create a compounding effect for both the child and family (30, 31) with lower levels of social support than the general population (32, 33).

It has been demonstrated that developmentally informed relationally focused intervention occurring following maltreatment can mitigate the negative impacts of maltreatment and developmental trauma for these children. Hambrick et al. (6) demonstrated how relationally positive experiences, attuned to an individual child's needs, can buffer the impact of the effects of maltreatment and improve relational health.

Toth et al. (34) explored the impact of child maltreatment on attachment and highlighted the importance of relational interventions for children and their families to address the negative consequences of maltreatment. They drew on Cicchetti (35) who explored the concept of allostatic load or the impact on the body when under major or sustained stress and how this effect on the body is likely to continue without intervention strategies to reduce the burden across the life span. Hart and Rubia (36) reviewed the abnormalities in the brain in maltreatment and adults with history of maltreatment highlighting the severity and complexity of the consequences of maltreatment.

### Evidence of Interventions for Maltreatment

Research has shown that adverse behavioral and emotional effects resultant from child maltreatment can be moderated by specific therapeutic interventions. A meta-analysis of an array of therapeutic interventions across 21 studies, totaling 964 participants, found 71% of children in treatment significantly improved compared to children in the waitlist, placebo or community case management control groups (37). Due to a moderate average weighted effect size of *d* = 5.4 (38), and because meta-analyses generally collapse across treatment methods, it can be challenging to derive clear clinical practice guidelines from this study. What this research does demonstrate, however, is solid evidence showing some interventions perform better than others.

One of these interventions, cognitive-behavioral approaches, have shown particular promise as an intervention for maltreated children. This includes trauma-focused cognitive behavioral therapy (TF-CBT) (39, 40), which is a sequence of psychological interventions to help children to re-examine their trauma experiences and practice more constructive ways of coping (41). Interventions include psychoeducation, cognitive-based coping skills and stress reduction techniques (42). In a review on the effectiveness of cognitive-behavioral therapies for child maltreatment, Fraser et al. (43) found adequate evidence to show that TF-CBT was most effective for children who had experienced sexual abuse, and that when provided in combination with parent-child cognitive behavior therapy (CPC-CBT), is particularly suitable for children who have experienced physical abuse.

### Relational Health Interventions

Other therapeutic approaches which demonstrate promising evidence include those which target foster parents. Foster parent training is generally based on the principle that foster parents are best placed to identify challenging behaviors and to encourage more adaptive coping strategies (44). Leading from Bowlby's (45) work on attachment theory, some interventions with foster parents focus on developing a secure relationship between foster parents and the children in their care. These studies have found promising decreases in behavioral challenges among children who have experienced maltreatment (46–48). This lends evidence for the effectiveness of relational approaches to ameliorating the impacts of childhood maltreatment by improving the relational health of these children.

Another approach with a relational focus, Multi-Systemic Therapy (MST), was originally intended for children in the juvenile justice system who presented with oppositional defiant and conduct disorders (49, 50). MST has since been adapted as an intervention for other issues, including child maltreatment. The main aim of MST is to understand how the child's difficulties fit within the systems surrounding the child, such as education or child protection systems (51). Given that the effects of child maltreatment are experienced across the child's social environments, MST aims to work collaboratively with these systems to tailor interventions across all of the areas where the child is exhibiting challenging behaviors or experiencing distress (51, 52). The ultimate goal of MST is the creation of a safe and caring social environment where maltreated children can, with support, develop more positive relationships with their family, friends and broader community (53).

Relational health is also addressed in other interventions approaches including Filial Family Therapy (54). Child-focused psycho-education (55), Child Parent psychotherapy (56), Eye Movement Desensitization and Reprocessing [EMDR; (57)], and Family Therapy (58) may be utilized solely or with a combination of other approaches to address the impact of maltreatment.

While there is a burgeoning of promising evidence in the literature on effective interventions for maltreated children, systematic reviews are yet to yield sufficiently strong evidence for any particular approach (43, 59). The research has also yet to be utilized specifically with an Australian Indigenous population or modified to ensure cultural appropriateness, which given the overrepresentation of Aboriginal and/or Torres Strait Islander children in out of home care in Australia, is a crucial step in building the evidence base for interventions with maltreated children.

### NMT as a Framework for Intervention Planning

There have been promising gains in the development of evidence-based frameworks for assessing the impact of maltreatment on a child's neurological and physiological development (17). Given the heterogenous nature of both the population of children who experience maltreatment, and the diverse range and nature of their maltreatment experiences there is not a one-size-fits-all intervention (60). The individual child's experiences and how these have impacted on that child's unique developmental trajectory need to be identified and a decision made as to which evidence-informed interventions would be the most suitable to address the challenges experienced by a particular child (61).

One framework for understanding and assessing the impacts of childhood maltreatment is the Neurosequential Model of Therapeutics (NMT) developed by the ChildTrauma Academy (17). The NMT is a neuroscience-informed, developmentally sensitive approach to clinical problem solving (62, 63). The NMT is based on extensive research and evidence on neurobiology, childhood development and the impacts of maltreatment and complex trauma on the developing brain (64). This model “helps match the nature and timing of specific therapeutic techniques to the developmental stage and brain region and neural networks mediating the neuropsychiatric problems” [(65), p. 38].

**Figure 1:**
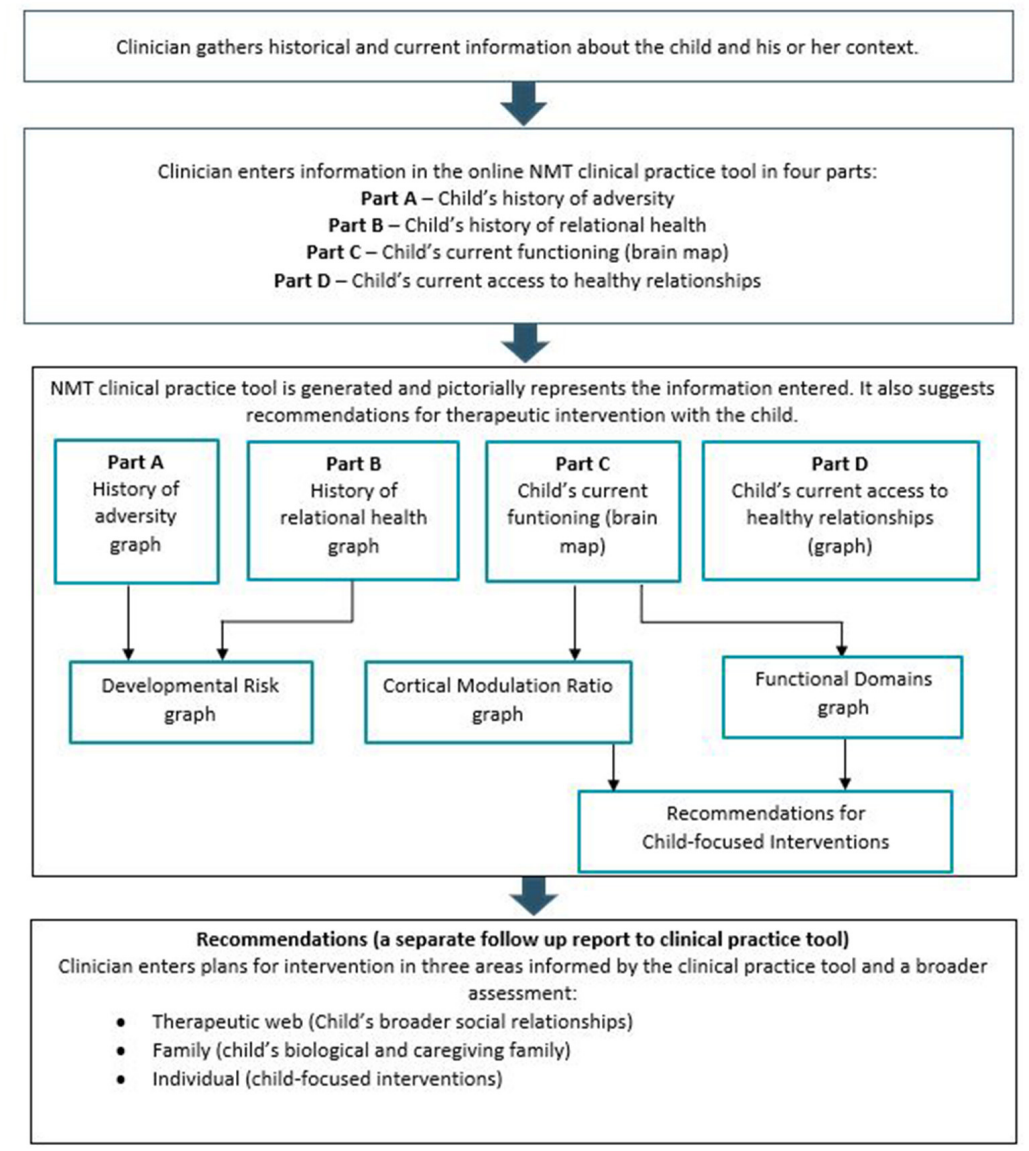
An overview of the NMT process. Jackson et al. (67).

A key goal of the NMT is to assist clinicians to formulate individualized intervention plans that consider the specific relational and developmental needs of the client (66). Information is gathered to populate key metrics, measuring the child's history of adversity and relationships, their current developmental functioning, their executive functioning capacity, and their current relational health. From this understanding, a range of formal and informal therapeutic interventions, activities and approaches are incorporated into an individually tailored treatment plan (see Figure 1). The NMT metric recommendations section provides a structured and systematic approach to planning and reviewing interventions at multiple levels (therapeutic web, family and individual). Within these levels, specific recommendations identify the priority level of the intervention (essential, therapeutic, or enrichment).

The NMT approach recognizes that regulation and resilience building need to occur within a context of adequate relational health (6). Where a child is assessed as having impoverished relational health, the NMT model prioritizes improving this as the first aim of intervention. The NMT clinical practice tool provides direction as to the type and timing of interventions that are most clinically indicated for the child (68).

### Relational Health

One of the key tenets of the NMT approach is the centrality of safe, enduring relationships as a precursor to both healthy normative development and to the treatment of the impacts of abuse and neglect (69). A maltreated child who is placed in a safe and stable, positive and caring placement may not experience these relationships as such, and Purvis and Cross (70) note the importance of differentiating “between a child actually being safe and feeling safe” (p. 363).

The NMT approach recognizes that regulation and resilience building need to occur within a context of adequate relational health (6). Where a child is assessed as having impoverished relational health, the NMT model prioritizes improving this as the first intervention.

For the purpose of this study, relational health refers to the number of connections a child has in their life as well as the quality and the lived experience of these connections for the child. Wheeler et al. (71) examined the effect between Adverse Childhood Experiences (ACEs) and indicators of mental and relational health. “ACEs include abuse, neglect, and household dysfunction ranging from a parent/household member with a mental illness to parental divorce” Wheeler et al. [(71); p. 24]. Wheeler et al. (71) found that ACEs were predicative of social impairments and lower overall relationship quality amongst adults. They recommended that “interventions be aimed at reducing overall relationship stress as a primary prevention strategy” (p. 26). Perry et al. (65) discussed the importance of interventions to commence strengthening the therapeutic web (i.e., number of healthy relationships to support the child's and family's social and emotional wellbeing) (65).

### The Importance of Relationally Positive Experiences

Two predictors of a child's current level of functioning are their current relational health and the level of developmental risk they experienced in their first two months of life (6). It is hypothesized that the brain's stress response system can be calmed by positive relational experiences, and when provided regularly and consistently this leads to increased opportunities for normative developmental growth (69). This highlights the crucial importance of relational health to the wellbeing and positive development of children who have experienced maltreatment, making it important to consider the level and nature of relational health in both placement decisions and clinical intervention plans.

Further, the finding that current relational health was a stronger predictor of current functioning than developmental risk, lends evidence to the position that children need to experience safe and positive relationships in order to thrive (69). For maltreated children with poor current relational health, the most effective intervention is to focus on the development and maintenance of relational health through positive connection to carers, peers, and community (69).

### Study Context

Berry Street's Take Two service is an innovative Australian biopsychosocial therapeutic service for infants, children and young people who have experienced serious abuse and/or neglect and are at risk of developing, or already present with, emotional and/or behavioral challenges. Take Two provides active outreach working with children, caregivers and the service system to build a network of positive relationships around the child. Take Two is a clinical service with research and training capacity embedded within the model and the service represents a good practice treatment model for children who have experienced maltreatment (61).

Take Two has operated since 2004 throughout Victoria, one of Australia's six states and its second most populated with about six million inhabitants. Berry Street, one of the largest independent child and family welfare organizations in Australia, is the lead agency for Take Two and partners with La Trobe University Discipline of Social Work and Social Policy, Mindful (Center for Training and Research in Developmental Health, University of Melbourne Department of Psychiatry) and Victorian Aboriginal Child Care Agency (VACCA). The Take Two staff group is comprised of a multidisciplinary team of allied health clinicians, including but not limited to, psychologists, occupational therapists, family therapists, and social workers.

The complex situations of infants, children and young people who have experienced severe maltreatment and childhood trauma experience particular challenges and needs that cannot always be addressed only by the more traditional clinical mental health services, or the social work based case management programs. Infants, children and young people who have experienced maltreatment, including neglect, during the developmental years are likely to have complex developmental disruptions which require multidisciplinary assessment and interventions beyond traditional individual therapy and psychiatric medication.

Relational and ecological approaches form the cornerstone of Take Two practice. Take Two's approach to therapy utilizes developmentally informed and trauma-focused intervention methods to assist children to make sense of their experiences and to prevent their past traumas from manifesting either intrusively through flashbacks and nightmares, or by causing them to utilize antisocial or self-destructive coping responses. At a relational level, Take Two practice is informed by attachment theory (72, 73) and includes coaching parents and/or carers to be able to respond empathically and constructively to children's developmental needs and disruptive behaviors. Improving the relational health of maltreated children is critical to support subsequent development, such as peer relationships, affect regulation and physiological regulation (74).

Take Two prioritizes the need for a culturally informed approach for all clients. This is especially important for Aboriginal and/or Torres Strait Islander children engaged in the service. The service has dedicated positions for Aboriginal staff members who provide cultural consultation and advice, as well as supporting clinicians to complete a cultural connectedness assessment and plan culturally sensitive intervention (75). The approach also has included changes/additions to the outcome measurement tools utilized to ensure they are culturally appropriate.

Finally, at a broader system level, Take Two works with the education system, the child protection system and any available social network in which the child participates, including sporting clubs, interest groups and extended family. Systems work can include advocacy, education or dyadic work, but whatever the precise intervention, the goal is always to support those around the child to be empathic, reflective, purposeful and therapeutic in their everyday interactions with the child. In the local context, a care team is frequently established consisting of the young person, parents, caregivers and professionals that are engaged in providing coordinated care. Take Two are active members of these care teams.

A suite of clinical measures has been implemented to canvass change across a depth and breadth of emotional and developmental functioning. Measures have been targeted toward age and stage, and include parent, caregiver, and teacher reports as well as self-reports for children over age eight and is aligned with the Take Two outcomes framework developed in 2004 (76).

Take Two selected NMT as an assessment and intervention planning framework to support clinicians to take a developmental trauma lens when working with referred children in addition to the methods described earlier.

Take Two is a flagship of the model in Australia and site-certified in the use of the NMT clinical practice tools, or “metrics” which provide a semi-structured assessment of important developmental experiences and a current “picture” of developmental functioning and the degree of positive connectedness to carers, family, peers and community (relational health) to guide intervention planning. The NMT Metrics assist Take Two clinical staff to select and sequence a range of evidence-informed clinical interventions including child psychotherapy, child-parent psychotherapy, family work, dyadic work, child-focused parent therapy, eye movement desensitization and reprocessing (EMDR), play therapy, art therapy, music therapy, somatosensory activities, case conferences, and psycho-education for parents or guardians.

Take Two characterizes itself as a relationship-based service. It recognizes that as the referred infants, children and young people are harmed within the context of relationships, their subsequent healing must also occur within their relationship network (24), which we refer to as the child's therapeutic web. As such, Take Two uses a systems approach to clinical work, beginning with a semi-standardized assessment protocol across multiple life domains, which includes completion of the NMT metrics. A goal and intervention plan is developed from this assessment, utilizing the NMT Metric recommendations section and this usually focuses on a range of psychological and social interventions. These interventions can be largely categorized into the NMT brain heuristic of regulate, relate and reason, which correspond to the three main brain areas—the brainstem, the limbic system, and the cortex (17).

The interventions are usually a combination of three empirically supported treatment methods with a combination of psychological and psychosocial interventions. The first of these is systems-level casework (77), aimed at wrapping around the child and young person the most constructive and supportive social environment possible. The second method is attachment-oriented interventions (78, 79) aimed at strengthening the security of attachment and the relationship between the caregiver/s and the child; and third, trauma informed psychotherapy with the child, focused on their understanding of and responses to their trauma experiences and building their capacity for emotional regulation.

## Materials and Procedures

The aim of the current study was to explore the impact of individually tailored neurodevelopmental and trauma-informed relational interventions on a cohort of Australian children who have experienced maltreatment using a mixed methods study. Ethics approval was received from La Trobe University HREC (04-131).

The study had two components. A cross sectional design which includes quantitative analysis of baseline and repeat clinical measure data with a cohort of children aged 2–11 years, who were clients of Berry Street Take Two between 2014–2019, was conducted utilizing SPSS.

The purpose of the quantitative analysis was to explore the changes to the child's behavioral and psychological functioning following intervention using the standard clinical outcome measures which Take Two administers at initial assessment, review (3 months for clients <5 years of age and every 6 months for clients more than 5 years of age), and at case closure.

The measures utilized in this study were:

The Strengths and Difficulties Questionnaire (SDQ), for people aged from 2 years, is a brief measure of strengths and difficulties that has parent, teacher and self-report (from age eight) versions. It is comprised of the following scales: emotional symptoms; conduct problems; hyperactivity; peer problems; prosocial behavior, and a Total Difficulties Score (80).

The Health of the Nations Outcomes Scale for Children and Adolescents (HoNOSCA) is a 15-item clinician rated measure of mental health functioning used in Australia, New Zealand, and the UK that measures the domains of behavior, impairment, symptoms, and social functioning. The HoNOSCA has been demonstrated to have good discriminative and concurrent validity, good face validity, and to be sensitive to change (81, 82).

The NMT Metric facilitates examination of current developmental functioning and help identify a child's strengths and vulnerabilities in 32 brain-related areas (e.g., abstract reasoning, fine motor control, and attention). Four major functional domains are derived from these: sensory integration, self-regulation, relational, and cognitive. The results help identify the urgency of need for intervention and repair based upon how far the individual's functional capabilities are from neurotypical. This need is scored as essential, therapeutic, or enriching [(62), p. 49]. This provides direction as to the focus of interventions. In addition, the NMT clinical practice tools examine the child's developmental history of adversity and relational health, and of current relational health.

The NMT current relational health (CRH) domain refers to the quality and availability of relationships to the child encompassing parents, caregivers, siblings, extended family, peers, school, clubs, therapists//support services and community/culture. Clinicians rate the quality of each of the child's current relational experiences from Poor (1–3), Episodic (4–6), Adequate (7–9) to Positive (10–12) with the sum of these items called the total Current RH score (66). The Current Relational Health (CRH) Total is a composite score of all of responses related to the quality and availability of each of the relationships mentioned above. It does not differentiate specific relationships but examines global relational health in the wider context of an individual's relationships. The living arrangements of Take Two clients frequently change as does the composition of household members, thus the subject of the rating may change from biological siblings at Time 1 to foster care siblings at Time 2 or vice versa. Due to this reduced stability, the Current Relational Health Total is a more reliable measure of current relational health than the composite subscales. The CRH Total score sits on a scale that rates the quality of this therapeutic web from Impoverished, to Adequate, to Enriched.

### Cross Sectional Study Method

**Figure 2:**
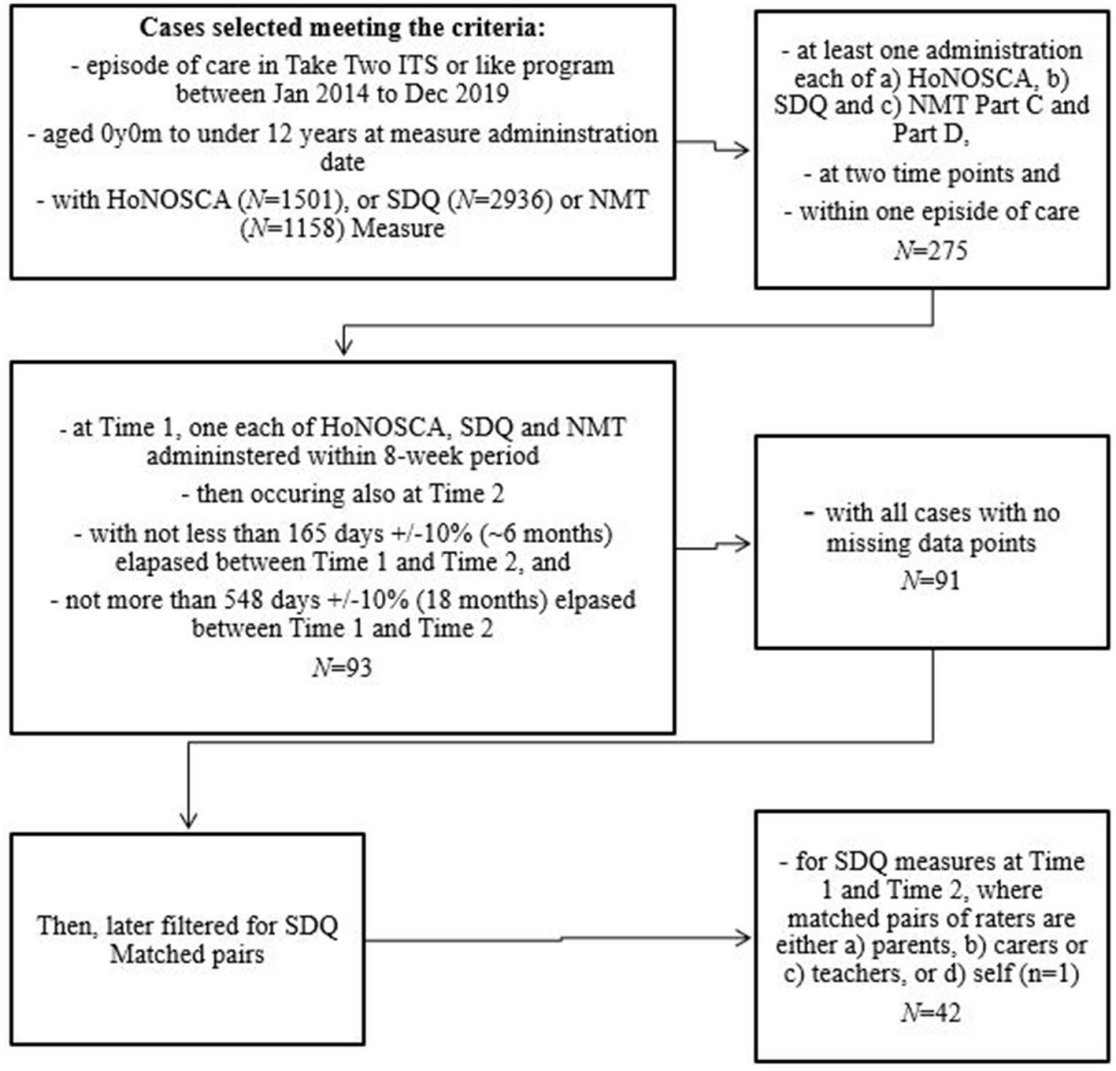
Sample selection flow chart.

A process of data selection (see Figure 2) was undertaken to determine the numbers of each quantitative measure available for analysis that complied with the desired sample criteria of receiving a Take Two service between 1st January 2014 and 31st December 2019, aged between 2 years, 0 months−11 years, 11 months, with a minimum of five months intervention, administration of the HoNOSCA, SDQ and NMT metrics within a six week period at two time points at least six months distant. Data cleaning, as described in Figure 2, led to a final study sample of *n* = 91. January 2014 was selected as the earlier cutoff date as this aligned with all three measures being administered and December 2019 was the end of the last full year prior to data analysis.

NMT metric reports completed as fidelity exercises, practice, or training, or those that contained missing or errors or client identifiable information were excluded. NMT Metric reports completed by clinicians with low fidelity also were excluded.

Descriptive data was obtained from referral documentation.

SDQ Attribute Scales (Emotional Symptoms, Conduct Problems, Hyperactivity, Peer Problems) and Total Difficulties) and HoNOSCA Scales (disruptive, antisocial or aggressive behavior; over activity, attention and concentration; scholastic or language skills; non-organic somatic symptoms; emotional and related symptoms; peer relationships; self-care and independence; family life and relationships; poor school attendance and total difficulties) were selected for analysis. This was based on findings of significance in previous analysis of Take Two interventions (76) and alignment with the target behaviors/symptoms of the intervention as described by Valentino (74) as relationships, affect regulation and physiological regulation.

A paired sample *t*-test was utilized to ascertain if there was significant change over time. A priori calculations anticipated that a desired effect size of 0.4 will be obtained from a sample size of 52 with power of 0.8. An effect size of 0.2 is generally considered small; 0.5, medium; and 0.8, large (38).

### Qualitative Method

The quantitative data analysis was supplemented by three case studies of Take Two clients. The purpose of the case studies was to add to understanding the process of intervention, including intervention type and timing. The case studies provided a more detailed explanation of the relational health interventions of Take Two and add to knowledge of the impact of interventions analyzed in the quantitative data presenting a real life context of the interventions (83).

The children selected for the case studies were aged between 2 and 11 years who had experienced maltreatment and received Take Two intervention for at least 5 months and were part of the quantitative sample. The selection was purposive to ensure sufficient data for analysis and excluded cases where the researcher's direct clinical supervisees had involvement. The researchers did not know the results of the clinical outcome measures when selecting the cases.

The case studies included analysis of case record material, to identify the types of interventions provided by Take Two following the NMT assessment as well as the impact of positive relational experiences and NMT-informed interventions on the psychological and relational health of these infants and young children.

A data collection tool was developed (see Appendix A), including demographic information, clinical measurement findings, length of intervention, number of sessions, intervention provided and frequency. The data collection tool was piloted with one case study and refined for use with the three selected case studies for analysis.

The data collection tool was completed for each of the case studies by three researchers and consultation was obtained from a Take Two Aboriginal clinician on the Aboriginal client case study. All researchers were engaged in the work of Take Two with extensive experience working therapeutically with children impacted by child maltreatment. All the researchers met to discuss their interpretation of material in a process of demonstrating construct validity and reliability.

## Results

The population for this study is a Take Two cohort of clients who received intervention between 2014 and 2019. The study sample (*n* = 91) was drawn from this population. Of the sample, 35.2% were female and 25.3% identified as Aboriginal and/or Torres Strait Islander. The average age was 7.4 years at Time 1 and 8.3 years at Time 2. The age range was 2.6 years to 11.9 years. The average treatment length was 324 days with a range of 155 days to 654 days.

Children in the sample at baseline were found to be experiencing difficulties in the clinical range on the following domains of the HoNOSCA: Overactivity, attention and concentration (*x* = 2.2); Emotional and Related Symptoms (*x* = 2.5), Peer relationships (*x* = 2.0) and Family Life and Relationships (*x* = 2.7). The degree of symptomology in the study sample at baseline was generally higher than both a European and an Australian Child and Adolescent Mental Health Services (CAMHS) cohorts (see Table 1). On the SDQ domains at baseline (see Table 2), the sample population was found to have high scores (substantial risk of clinically significant problems) in the Conduct domain and Total Difficulties and slightly raised scores (may reflect clinically significant problems) in the domains of Hyperactivity and Peer Problems (84). The study sample baseline scores were also higher on every domain than normative samples from Australia and the USA (see Table 2).

**Table 1:** Baseline HoNOSCA mean scores (*n* = 91).

**HoNOSCA scales**	**Study sample *n* = 91**	**Norwegian CAMHS population^**a**^ (*n* = 153)**	**Australian CAMHS outpatient population (*n* = 15,000) aged 7–10 years, 2014–2019 at admission^**b**^**
	***$\bar{x}$***	***$\bar{x}$***	***$\bar{x}$***
Disruptive, antisocial, or aggressive behavior	1.9	1.2	1.9
Overactivity attention and concentration	2.2^*^	1.9	1.8
Scholastic or language skills	1.8	1.5	1.3
Non-organic somatic symptoms	0.8	0.5	0.9
Emotional and related symptoms	2.5^*^	1.6	2.5^*^
Peer relationships	2.0^*^	1.5	1.8
Self-care and independence	0.9	0.4	0.7
Family life and relationships	2.7^*^	1.5	2.2^*^
Poor school attendance	0.4	0.5	0.9
Total difficulties	16.0	12.0	14.8

a *Hanssen-Bauer et al. (81)*.

b *https://www.amhocn.org/nocc-reporting/amhocn-data-portal*.

* *Clinically Significant Range = 2–4. Australian Mental Health Outcomes and Classification Network (84)*.

**Table 2:** Baseline SDQ mean scores (*n* = 91).

	**Study sample (*n* = 91)**	**Australian normative sample^**a**^*N* = 197 parent report, males aged 7–10 years**	**USA normative sample^**a**^*N* = 2,064, aged 8–10 years**
	***$\bar{x}$ (SD)***	***$\bar{x}$ (SD)***	***$\bar{x}$ (SD)***
Emotional symptoms	3.88 (2.61)	2.3 (2.1)	1.5 (1.9)
Conduct problems	4.51^*^ (2.60)	1.8 (1.7)	1.3 (1.7)
Hyperactivity	6.46^**^ (2.69)	4.1 (2.7)	2.9 (2.6)
Peer problems	3.45^**^ (2.24)	1.8 (2.0)	1.5 (1.6)
Total difficulties score	18.30^*^ (7.13)	9.9 (6.4)	7.2 (5.8)

* *This score is high: substantial risk of clinically significant problems*.

** *This score is slightly raised, may reflect clinically significant problems*.

a *https://www.sdqinfo.org/g0.html www Youth in Mind*.

Analysis of repeat measures at Time Two found children demonstrated statistically significant improvement as measured by the HoNOSCA (*n* = 91) in particular in relation to their Total Difficulties (*p* = < 0.01), disruptive, antisocial or aggressive behavior (*p* = < 0.01), over activity, attention and concentration (*p* = < 0.01), non-organic symptoms (*p* = 0.02); emotional and related symptoms (*p* = < 0.01); peer relationships (*p* = < 0.01), family life and relationships (*p* = < 0.01) as well as caregiver understanding of difficulties (*p* = 0.04). Effect sizes were calculated using Cohen's d with Hedges correction with small to medium effect sizes (0.2–0.6) found on all domains, except poor school attendance (see Table 3).

**Table 3:** Change over time as measured by the HoNSOCA (*n* = 91).

	***$\bar{x}$ (SD)***				**Inferential statistics**	
	**Time 1**	**Time 2**	**Change in score**	**Hedges *g***	***t-*test**	***p*-value**
Disruptive, antisocial or aggressive behavior	1.85 (0.98)	1.46 (0.92)	0.389	0.4	4.262	0.000
Over activity attention and concentration	2.16 (0.99)	1.79 (1.05)	0.368	0.3	3.445	0.002
Scholastic or language skills	1.81 (1.17)	1.59 (1.14)	0.221	0.2	2.106	0.056
Non-organic somatic symptoms	0.78 (1.12)	0.55 (0.92)	0.232	0.3	2.347	0.020
Emotional and related symptoms	2.46 (0.92)	2.09 (0.889)	0.368	0.4	3.380	0.001
Peer relationships	1.95 (1.04)	1.63 (1.04)	0.316	0.3	2.891	0.004
Self-care and independence	0.85 (0.91)	0.71 (0.84)	0.147	0.2	1.862	0.066
Family life and relationships	2.63 (0.99)	2.28 (0.94)	0.347	0.4	3.549	0.000
Poor school attendance	0.34 (0.75)	0.41 (0.90)	−0.074	−0.1	−0.961	0.408
Total difficulties	15.61 (5.77)	12.95 (6.23)	2.663	0.6	5.426	0.000
Knowledge of nature of difficulties	1.33 (1.07)	1.05 (1.08)	0.274	0.2	2.092	0.039

The SDQ data set in this study consisted of measures completed at Time 1 by parents (*n* = 12), carers (*n* = 39), teachers (*n* = 38), self (*n* = 1), and other (*n* = 1) and at Time Two by parents (*n* = 16), carers (*n* = 44), teachers (*n* = 26), self (*n* = 2), and others (*n* = 3). Analysis of all SDQ data found change in a positive direction on Total Difficulties (*p* = 0.08) and a statistically significant improvement in the domain of Peer Problems (*p* = 0.05). Small effect sizes (0.2) were found for Total Difficulties, Peer Problems and Conduct Problems (see Table 4).

**Table 4:** Change over time as measured by the SDQ—all respondents (*n* = 91).

	***$\bar{x}$*** **(** ***SD*** **)**		**Change in score**	**Hedges *g***	**Inferential statistics**	
	**Time 1**	**Time 2**			***t*-test**	***p-*value**
Total difficulties score	18.30 (7.13)	16.95(7.74)	1.35	0.2	1.77	0.08
**SDQ subscales**						
Emotional symptoms	3.88 (2.62)	3.47 (2.57)	0.41	0.1	1.36	0.18
Conduct problems	4.51 (2.60)	4.00 (2.41)	0.51	0.2	1.68	0.10
Hyperactivity	6.46 (2.69)	6.46 (2.62)	0	0	0.00	1.0
Peer problems	3.45 (2.42)	3.01 (2.19)	0.44	0.2	1.96	0.05

The SDQ repeated data set was recoded for matched respondent pairs (*n* = 42). The data set of all matched respondent pairs was analyzed (see Table 5) with a significant change over time found on both the Peer Problems (*p* = 0.05) and Total Difficulties domains (*p* = 0.03). Further analysis conducted on each respondent grouping found parents (Table 6; *n* = 5) reported significant change on peer problems (*p* = 0.05) and Total Difficulties (*p* = 0.02) with carers (Table 7; *n* = 24) also reporting significant change on peer problems (*p* = 0.05). Teacher completed SDQs (Table 8; *n* = 12) did not reveal significant change over time on any domain.

**Table 5:** Change over time SDQ completed by matched pairs different respondents (*n* = 42).

	**Time 1 *$\bar{x}$ (SD)***	**Time 2 *$\bar{x}$ (SD)***	**Change in score**	***t*-test**	***p*-value**
Emotional symptoms	4.02 (2.5)	3.6 (2.45)	0.42	1.01	0.32
Conduct problems	4.67 (2.58)	3.90 (2.49)	0.77	1.88	0.067
Hyperactivity	6.69 (2.60)	6.38 (3.09)	0.31	0.85	0.399
Peer problems	3.55 (1.91)	2.83 (2.06)	0.72	2.82	0.007
Total difficulties	18.93 (7.09)	16.71 (8.38)	2.22	2.25	0.030

**Table 6:** Change over time SDQ completed by matched pairs—parents (*n* = 5).

	**Time 1 *$\bar{x}$ (SD)***	**Time 2 *$\bar{x}$ (SD)***	**Change in score**	***t*-test**	***p*-value**
Emotional symptoms	4.0 (3.65)	3.40 (2.89)	0.60	2.06	0.11
Conduct problems	3.80 (1.48)	2.00 (1.23)	1.8	2.45	0.07
Hyperactivity	4.80 (1.79)	4.60 (2.30)	0.2	0.34	0.75
Peer problems	4.00 (1.00)	2.60 (2.07)	1.4	2.75	0.05
Total difficulties	17.20 (5.45)	12.60 (7.30)	4.6	3.37	0.03

**Table 7:** Change over time SDQ completed by matched pairs—carers (*n* = 24).

	**Time 1 *$\bar{x}$ (SD)***	**Time 2 *$\bar{x}$ (SD)***	**Change in score**	***t*-test**	***p*-value**
Emotional symptoms	4.38 (2.23)	3.71 (2.33)	0.67	1.08	0.29
Conduct problems	5.08 (2.54)	4.21 (1.91)	0.87	1.73	0.10
Hyperactivity	6.92 (2.59)	6.71 (3.00)	0.21	0.42	0.68
Peer problems	3.58 (1.98)	2.96 (2.01)	0.62	2.04	0.05
Total difficulties	19.96 (7.06)	17.58 (7.52)	2.38	1.84	0.08

**Table 8:** Change over time SDQ completed by matched pairs—teachers (*n* = 12).

	**Time 1 *$\bar{x}$ (SD)***	**Time 2 *$\bar{x}$ (SD)***	**Change in score**	***t*-test**	***p-*value**
Emotional symptoms	3.33 (2.46)	3.58 (2.78)	−0.25	−0.32	0.75
Conduct problems	4.42 (3.03)	4.42 (3.37)	0	0.00	1.00
Hyperactivity	7.25 (2.70)	6.92 (3.20)	0.33	0.44	0.67
Peer problems	3.50 (2.11)	2.92 (2.23)	0.58	0.94	0.37
Total difficulties	18.50 (7.60)	17.83 (9.82)	0.67	0.30	0.77

The NMT Current Functioning domain mean scores were re-coded as a percentage of age typical to accommodate for developmental growth. Change over time was calculated using the percentage of age typical means for each current functioning domain. Children were found to achieve statistically significant improvements on all Current Functioning domains (see Table 9), Sensory Integration (*p* = < 0.01), Self-Regulation (*p* = < 0.01), Relational (*p* = < 0.01), and Cognitive (*p* = 0.02). Small to medium effect sizes were found on all Current Functioning domains (see Table 9). A significant positive change over time was also found on the NMT measure of Current Relational Health (CRH) total (*p* = < 0.01) with an effect size of 0.4 (see Table 10).

**Table 9:** Change over time as measured by the NMT current functional domains.

	**% age typical** ***$\bar{x}$*** **(** ***SD*** **)**		**Change in score**	**Hedges *g***	**Inferential Statistics**	
	**Time 1**	**Time 2**			***t*-test**	***p-*value**
Part C sensory integration	0.86 (0.12)	0.89 (0.10)	0.03	0.4	−3.90	0.00
Part C self-regulation	0.78 (0.11)	0.81 (0.10)	0.03	0.5	−4.42	0.00
Part C relational	0.77 (0.11)	0.81 (0.11)	0.04	0.6	−5.75	0.00
Part C cognition	0.81 (0.14)	0.83 (0.12)	0.02	0.3	−2.40	0.02

**Table 10:** Change over time as measured by the NMT current relational health total.

	***$\bar{x}$ (SD)***	***$\bar{x}$ (SD)***	**Change in score**	**Hedges *g***	***t*-test**	***p-*value**
Current relational health total	56.30 (13.29)	61.71 (12.55)	−5.41	0.4	−4.16	0.00

In summary, the analysis showed changes in the strength of the child's relational health was significant (*p* = < 0.01; cohen's *d* = 0.4) and changes in all NMT current functioning domains were significant (cohen's *d* = 0.3–0. 6) as well as peer relationships (*p* = < 0.01; cohen's *d* = 0.3) and family relationships (*p* = < 0.01; cohen's *d* = 0.4), disruptive behavior (*p* = < 0.01; cohen's *d* = 0.4) and emotional symptoms (*p* = < 0.01; cohen's *d* = 0.4) as measured by HoNOSCA. Significant improvements were also reported by carers on the SDQ analysis in peer problems (*p* = 0.05; cohen's *d* = 0.2) and total difficulties (*p* = 0.03).

### Case Studies

Three case studies are presented here as vignettes to provide a real-life view of the work of Take Two clinicians in intervening to strengthen relational health of the child client. As will be seen each case presents a different scenario and thus different types of intervention. However, each intervention sought to build a common and informed understanding of the child between adults in the child's therapeutic web and improve relational health. The material has been de-identified and pseudonyms used.

## Case Vignette 1

Ken was 4 and a half years old when he was referred to Take Two by Child Protection experiencing problems with sleeping and eating. Ken was born substance dependent and at the time of referral to Take Two he was living with his grandmother and she worked hard to look after him. Prior to the referral to Take Two, his grandmother had some problems with hoarding and child protection were concerned that clutter in the home posed a safety risk for Ken. He was removed temporarily from his grandmother's care for five days and during this period he spent time in three different homes as well as extended periods of time in a Child Protection or Foster Care agency office and did not see his grandmother or other attachment figures. Ken was returned to his grandmother's care after 5 days and was subsequently referred to Take Two with concerns about his emotional reactivity (outbursts like a much younger child), anxiety and withdrawn presentation and received an 11-month intervention.

### Intervention Plan

Following assessment Take Two recommended child-focussed psycho-education sessions with Ken's grandmother to support her understanding of his presenting issues and to develop strategies to manage them. Dyadic sessions with Ken and his grandmother were also recommended, with the aim of developing a shared narrative about Ken's removal from his grandmother's care and to repair the rupture that had occurred in their relationship as a result of the removal. As with all Take Two clients, regular meetings with K's care team was also a recommendation.

### Interventions

#### Child and Family Interventions

Over an 11-month period Take Two provided a mixture of child-focussed psychoeducation sessions to Ken's grandmother alongside some dyadic sessions. During her sessions Ken's grandmother was able to acknowledge her feelings of guilt around the removal of Ken from her care, her fear that it would happen again and that these feelings were getting in the way of her being able to be an attentive and playful carer for Ken. This informed the dyadic work, with play being the focus as well as supporting the dyad to communicate about emotions and needs.

#### Systems Interventions

The clinician maintained contact with those in Ken's therapeutic web which included the kindergarten, Child Protection staff, the care team and with the health service where Ken's grandmother was receiving treatment. This included advocating for an increased understanding of Ken's difficulties and the implementation of strategies to meet his needs.

#### Closure and Outcome

At the conclusion of the intervention Ken had made developmental gains in the areas of feeding, sleeping and emotional regulation though he was still below age typical. Ken's grandmother reported feeling more relaxed about her caring role and was enjoying the play activities suggested by the Take Two clinician. Although the intervention did not produce a coherent narrative about Ken's removal from his Grandmother's care there was an increase in the dyad's capacity to communicate about how they were feeling and what would help each other to feel better. Ken's grandmother's hoarding issues were still present, but she was seeking psychological support to manage these.

The NMT Current Relational Health score reflects this improvement, changing from 40 at the beginning of the episode, to 59 at closure. Ken's overall self-regulation and relational functioning showed improvements also—the former from 82% age typical to 89%, the latter from 74 to 80%.

These improvements in Ken's relational health are reflected in both his improvements in the NMT domains of Self-Regulation and Relational Functioning, controlled for age, and in Carer reported improvements in Ken's presentation in the home environment.

On Total Difficulties and three of the domains (Emotional Symptoms, Conduct Problems and Peer Problems) of the Carer completed SDQ, Ken moved from having his difficulties described as being in the Significant Difficulties category (substantial risk of clinical problems) to Minor Difficulties by the end of the intervention. On the domain, Hyperactivity, Ken changed from displaying Some Difficulties to Minor Difficulties (see Table 11).

**Table 11:** Case vignette summary of changes in clinical measures over time.

	**Ken**		**Xavier**		**Clinton**	
**Gender**	**M**		**F**		**M**	
**Time**	**T1**	**T2**	**T1**	**T2**	**T1**	**T2**
Age at Administration (years)	4.4	5.1	5.1	5.6	9.0	10.0
HoNOSCA total difficulties	12	9	15	13	24	16
SDQ rater	C	C	C	C	T	P
Emotional symptoms	6	2	3	4	6	3
Conduct problems	4	1	9	7	0	3
Hyperactivity	6	3	5	9	8	5
Peer problems	5	1	3	4	0	4
SDQ total difficulties	21	7	20	24	14	15
**NMT**						
Part C cognitive % age typical	0.80	0.90	0.80	0.72	0.72	0.82
Part C relational % age typical	0.74	0.80	0.77	0.72	0.77	0.91
Part C sensory integration % age typical	0.86	0.88	0.97	0.89	0.84	0.89
Part C self-regulation % age typical	0.82	0.89	0.84	0.81	0.77	0.87
Part D current relational health total	40	59	43	46	59	63

## Case Vignette 2

Xavier is a 6-year-old Aboriginal boy referred to Take Two by Child Protection, due to concerns about the stability and safety of his current living situation. Xavier was displaying extremely dysregulated behaviors on an almost daily basis—head banging, pulling out his hair, kicking, punching others, and scratching himself. He had also harmed animals. He had poor sleep, poor academic performance, and hyperactivity.

At the time of referral to Take Two he lived with a family friend (Anna, his primary caregiver), Anna's infant daughter and Anna's aunt Linda. Xavier had been removed from his parents' care at the age of 3 years due to concerns about his mother's deteriorating mental health and substance use. Xavier was placed with Anna who he had known since birth and who had often babysat and cared for him.

The clinical formulation noted the profound impact of prolonged exposure to family violence and unpredictable caregiving on Xavier's functioning. In particular, he was reactive to unpredictability and change, and he struggled to rely on his primary caregiver who was unable to consistently respond to his underlying needs for co-regulation and developmentally-attuned care.

### Intervention Plan

Following the assessment, the clinician recommended Filial Therapy (54) for Xavier and Anna, alongside carer psychoeducation sessions. Filial therapy is an evidence-informed child centered relationship enhancement family therapy in which “parents learn to conduct child-centered play sessions with their own children” (85): (1). Anna was unprepared to engage in the Filial Therapy, but was prepared to engage with the clinician to gain a better understanding of the reasons why Xavier might be behaving in these worrying ways, and to develop strategies to respond to the behaviors with the aim of reducing their frequency and intensity.

The primary goals of the carer psychoeducation were to:

- Address Xavier's poor sleep by developing a predictable, consistent and relationally rewarding night-time routine between Xavier and Anna.
- Address Xavier's sensitized stress neurobiology, manifested in his extreme behaviors, by developing a more predictable, structured household routine that allowed times for Xavier and Anna to connect, as well as opportunities for Xavier to experience consistent caregiving and attuned responses to co-regulate him in a developmentally-targeted way.

### Interventions

#### Child and Family Interventions

Take Two provided a 9-month episode of care to Xavier. The direct therapeutic intervention involved six, one-hour sessions between clinician, Anna and sometimes, Linda. These psychoeducation sessions focussed on attachment-based concepts and models such as Circle of Security, including time in (86), relational repair (87), co-regulation (88), and Dan Hughes' PACE approach (89). Additionally, the clinician called Anna and Linda in the week prior to the session, so they could update her on Xavier's progress and any challenges they wanted to focus on in the upcoming session.

#### Service Systems Interventions

Take Two's participation in Xavier's care team (five meetings) was key to the intervention—ensuring that the key adults and decision-makers in Xavier's life shared a common understanding of his needs, and how to support Anna and Linda in their care of Xavier. This included the clinician's participation in Xavier's school Student Support Group meeting. The clinician also made monthly (six) phone calls to the Aboriginal Community Controlled Organization case manager to enable her to reinforce the psychoeducation in her own contact with Xavier, Anna and Linda.

### Closure and Outcome

The Take Two engagement ceased when Xavier's primary presenting behaviors of concern had decreased (his head-banging had ceased) and were having less of an impact on his relationship with Anna and Linda. Anna and Linda had demonstrated capacity to respond to Xavier in more relationally-focussed ways, reducing the co-dysregulation that had previously been a feature of their interactions.

## Case Vignette 3

Clinton is an 11-year-old girl referred to Take Two at the age of 9 years. At the time of referral Clinton was being reunited to live with her parents following a period of living in out of home care. Since her birth, Clinton had been placed in out of home care five times due to family violence between her parents and their substance use resulting in neglect of her and her brother. Prior to her current return to her parents, Clinton had taken responsibility for the care of her brother who was 3 years younger than her, preparing him for school and ensuring he had something to eat. When Clinton returned to her parents' care, the parents were separated, with the plan for her to spend half the week with each parent. However, Clinton's father became ill and was not able to care for her. Clinton's father only attended the first session with the Take Two clinician.

Clinton was referred to Take Two to support her strengthening relationship with her parents. At time of referral she presented with complex behavioral problems including parentified behavior toward her younger brother and aggression to her mother, rejecting her parenting role.

The assessment was undertaken with a developmental focus and included completion of the NMT, and other measures. On the NMT metric, Clinton's CRH was in the Adequate range. In the assessment of her current developmental functioning, her self-regulation score was 77% of age typical, as was her relational functioning.

### Intervention Plan

The objectives of the Take Two intervention included educating Clinton's parents on the impact of trauma, how to respond to her behavior, assisting her to self-expression in healthier ways and to feel secure and safe with her parents. To meet these objectives the intervention plan was as follows:

1. Systems intervention: participating regularly in a combined parents and professionals care team to establish a safe respectful and non-blaming space to share information, discuss progression of supports and identify concerns over time. Take Two provided psychoeducation to the care team drawing on findings from the clinical assessment to promote a common understanding of and response to Clinton's needs and functioning.
2. Child and Family Interventions. Child focused parent work, including psychoeducation.
3. Individual therapeutic work for Clinton to address her experience of trauma and find a more adaptive means to address the impact of her past traumatic experiences.

#### Interventions

The three intervention strategies were integrated over a 12-month period. The focus of the systems intervention was sharing information between professionals ensuring a common understanding of the issues facing Clinton and the desired response of all professionals. The Care team meetings engaged Clinton's mother to join with the professionals in ensuring her understanding of Clinton's needs. The 15 individual sessions utilized EMDR (57) including desensitization work focusing on Clinton healing from trauma and feeling safe and thus reducing her parentified behavior and aggressive response to her mother. In addition, Clinton's mother was engaged in part of Clinton's individual sessions. In these dyadic engagements the clinician modeled appropriate ways to interact with Clinton, and her mother could build new skills in engaging Clinton by guided participation in the early part of the sessions.

### Closure and Outcome

At the conclusion of the intervention the clinician noted changes in the mother's engagement with Clinton and in her enjoyment playing with Clinton and her level of attainment was notably higher than when intervention began.

Clinton's NMT self-regulation functioning was 87% age typical, and her relational functioning 91% and the family were coping as a family. This improvement in Relational Health is reflected in Clinton's improvement on the HoNOSCA Total Difficulties from 24 to 16 and on her Parent rated SDQ that describes her Emotional Symptoms as having moved from Significant Difficulties to Minor Difficulties and Hyperactivity from Significant Difficulties to Some Difficulties (see Table 11).

### Summary of Changes in Outcome Measures

Table 11 presents the outcomes data for the three case vignettes for HONOSCA, SDQ and NMT. It can be seen that Case 1 and 3 improved on all metrics of the NMT, whilst Case 2 age typical on NMT measures were lower, with the exception of relational health, which showed a slight improvement.

## Discussion

This study established that individually tailored neurodevelopmental and trauma-informed relational interventions contributed to significant improvements in children's relational health as well as behavioral and psychological wellbeing. This is consistent with Valentino (74) and Toth (34) who both discussed restoring children's regulatory capacity as an important measure of an effective treatment.

The case studies illustrated the role of integrated interventions delivered with the child and the therapeutic web. This is especially pertinent for children with maltreatment experiences as they are often experienced within the intersectionality of family violence, disability and reduced social supports (74). Thus, single interventions may not address the complex situations and factors impacting on a young person's presentation.

In the case studies the intervention plan was informed by the assessment and use of NMT and was influenced by the child's situation and their age. Each intervention utilized a trauma informed developmental lens, however, the type of intervention differed based on the child developmental stage and the family and system's understanding of the ecological system for the child. For Clinton, the oldest child in the vignettes, there was a focus on individual therapy integrated with dyadic treatment with her mother and systems intervention. For Ken in case vignette 1, the focus was upon child focused psychoeducation and dyadic work with the Grandmother, his primary carer. This assisted the Grandmother to address her own feelings of guilt of having the child removed from her care and to develop a more informed understanding of Ken's needs so that she could respond more appropriately. It was noted that during this time Ken made developmental gains although was still below age typical. Case vignette 2 highlighted the complexity of the impact of trauma and the often-slow progress of change. Moreover, as highlighted by the Aboriginal consultant more work could have occurred with the child's broader Aboriginal community and engaging in culturally informed interventions. The slight improvement in relational health demonstrates the importance of working with the therapeutic web to strengthen the supports available to the child. The case vignettes demonstrate the integration of individual, family and systems approaches which promote relational health.

### Strengths and Limitations

Strengths of this study include a robust sample size for the cross-sectional cohort study design allowing effect size to be determined. Review of similar studies examining relational health and NMT were significantly smaller (90, 91). The use of validated outcome measures to determine evidence of change following intervention for the study cohort is also a strength of the study. To our knowledge this is the first time a study examining relational health and utilizing NMT on this scale has been completed. The analysis of the measures allowed for the comparison between the Take Two sample and a normative sample (NMT). A limitation of the current study was that the design did not allow for comparison with another intervention type or a control group.

The use of multiple outcome measures (HoNOSCA, SDQ and NMT) allows for gathering a comprehensive understanding of the child's presentation from multiple respondents who reflect on the child's presentation in different settings, including both the impact of adverse events as well as the impact of intervention. In this cohort the SDQ reflected parents and carers' assessment that their children made significant gains in the home environment, however, in the school environment teachers noted smaller degrees of change (see Table 8).

A limitation of the study is that frequently the respondent completing the measure at Time 1 was not the same individual who completed it at Time 2. Reasons included change of clinician, class teacher, or a change of home circumstances which could lead to a change of primary carer or school. This reflects the complexity of these children's lives and the high level of change which can occur for them whilst they are receiving Take Two intervention. This is a challenge in undertaking research in this area (6).

In interpreting the changes between Time 1 and Time 2 in the clinical measures it is important to take into account that at Time 2 for the clinician, and perhaps other members of the therapeutic web, there is a deeper level of knowledge of the child and their environment. It is not known if this greater knowledge contributes to a changed interpretation of a situation. This may affect the scores reported in the clinical measures at Time 2.

It is not possible to identify change in specific relationships the child has with others and within the therapeutic web, from the outcome measures. The clinical measures do not allow for specificity of information regarding the impact of an intervention targeting specific relationships. However, reviewing the findings from the three measures indicates the elements which have changed in the child's presentation.

Another limitation is that only one of the measures includes self-report by the child and key members of the child's environment. The SDQ was the only measure that allowed for self-reports in this study, and it is noted that only three self-reports by children were completed. However, it is noted that the study cohort were all aged below 12 years and the Self report commences at age 11 years.

A limitation of the study is that the data for the case studies in this research were based on case records and did not include interviews with the child, clinician or other members of the child's environment.

The reduced stability in the living arrangements and household composition for the individuals in the study cohort limited the ability to determine if there were improvements in specific relationships contributing to the overall improvement in relational health. Refinement of data collection tools enabling recording of the specific relationship quality and availability being measured would achieve greater specificity in both intervention planning and outcome monitoring.

A next step in research in relation to the impact of Take Two would be a comparison of Take Two intervention with an alternative intervention of comparable duration and intensity. Whilst the study findings report positive changes in the children's presentations, the methodology did not include a comparison with an alternative intervention or usual care thus it is not possible to determine the outcomes were a direct result of the intervention.

Future research could compare the Take Two intervention with an alternative intervention and include in depth case studies including interviews with child, parents, carers, schools and clinicians and members of the therapeutic web. A qualitative study which included interviews with the child and key participants of the therapeutic web would provide more information of the process of the intervention which would supplement the quantitative analysis.

### Contextualization of Findings

The results of this study provide further evidence of the importance of relational health interventions to address developmental delay and trauma associated with experiences of maltreatment. Hambrick et al. (6), Ludy-Dobson and Perry (69), Purvis and Cross (70), and Toth (34) highlighted the role of relational health in achieving positive outcomes for children impacted by maltreatment. This study demonstrated the changes which occurred in the child's functioning when relational health was stronger. The improved functioning which was identified in the HoNOSCA and SDQ suggests further evidence of the impact of developmentally and trauma informed relational interventions.

The findings were consistent with those of an RCT (*n* = 120) of the ABC model, a brief relational intervention (92). The ABC study found that with children aged 6 months−2 years, who were living with their parents but involved with child protection services, there were positive outcomes in secure attachment (*d* = 0.38) and reduced disorganized attachment [*d* = 0.52; (93)]. All of the children were living with their parents. Our study showed comparable improvements in family life and relationships (*d* = 0.4), HoNOSCA total difficulties (*d* = 0.6), NMT Part C Domains of self-regulation (*d* = 0.5), and Relational (*d* = 0.6) functioning but with a larger age range, smaller sample, and different study design.

The Take Two assessment requires the clinician gather information about the child's life to input into the NMT metrics. The analysis undertaken provides feedback on the areas of the child's functions and life that is affected. The NMT assessment, then, provides the clinician and educator with the individual child's strengths and vulnerabilities in an array of key domains of functioning: sensory integration, self-regulation, relational, and cognitive (6). This information helps direct the selection and timing of developmentally appropriate enrichment, educational and therapeutic activities. Zarnegar et al. (91) and Barfield et al. (90) are studies evaluating programs utilizing NMT to guide intervention planning when working with maltreated children. Both found significant positive change with moderate to large effect sizes in the social-emotional development of the children, but with small sample sizes (*n* = 10; 13; 15) with the need noted for larger studies with a broader age range to evaluate the efficacy of NMT informed interventions, as has occurred with this study (*n* = 91) of children aged 2–11 years.

Mancini (94) found a significant positive change and a large effect size (*d* = 1.1) when evaluating a school based study (*n* = 34) utilizing a somatic soothing program and incorporating EMDR with a subset of the participants, suggesting future studies would be enhanced by the inclusion of teacher and family psychoeducation in the treatment process to enhance outcomes for participants. In case study 3 (Clinton) EMDR was utilized as one of the NMT recommended interventions together with active involvement from his mother in the EMDR sessions alongside the provision of psychoeducation contributing to improvements in Clinton's Relational Health and a reduction in her emotional symptoms from Significant Difficulties to Minor Difficulties.

Topitzes et al. (95) examined outcomes for children (*n* = 598) receiving trauma informed program services in the USA, incorporating NMT, with a focus on measuring the impact of the interventions on placement stability, reunification, child protection case closure or further maltreatment, including a comparison group. Importantly this study found a positive impact on placement permanency for the study cohort and is an area for further investigation in an Australian context.

Child Parent Psychotherapy (CPP) utilizes a similar intervention period to that used in our study and a smaller cohort (*n* = 65) but did utilize a comparison group and has been associated with decreased behavioral problems (*d* = 0.24) but at a lower effect size than in our study.

The SDQ was utilized as an outcome measure in a UK study (96) conducted with a CAMHS population presenting with Obsessive Compulsive Disorder (*n* = 31) achieving a similar small effect sizes on the SDQ Total Difficulties Scale, however on a more specific OCD measure, (The Short Obsessive Compulsive Scale), a large effect size (*d* = 0.9) was found. Lee et al. (96) recommended the use of at least two outcomes measures in future studies with one broad outcome measure to allow comparison with other studies but which tend to produce smaller effect sizes, and a more specialized measure to increase power to detect change. In this study we used the SDQ (a broad measure) and the HoNOSCA (a more specialized measure) with the SDQ results achieving lower effect sizes than the HoNOSCA, as predicted by Lee et al. (96) and Ford et al. (97). Efficacy studies usually exclude children with co-morbid conditions or intersectionality and utilize measures focussed closely on the target problem and thus the studies and results may not easily translate to services such as publicly funded CAMHS or Take Two, where children with comorbid conditions or intersectionality or not excluded (97, 98).

The findings of the quantitative analysis of the functioning of the child at baseline of the study highlights the severity of the impact of maltreatment on the study group. Consistent with Boon et al. (99) who studied outcomes for clients (*n* = 12,547; *d* = 0.5) of 10 child mental health facilities in The Netherlands, an effect size of 0.60 (cohen's *d*) was found in this study with a similar treatment length. The cohort used in this study did exhibit more severe symptomology at baseline as measured on all HoNOSCA Scales except for Poor School Attendance, with four items in the Clinically Significant Range (Over-activity, attention and concentration; Emotional and Related Symptoms; Peer relationships and Family Life and Relationships) compared with Norwegian [*n* = 153; (81)].

The study contributes to understanding the importance of addressing the pervasive impact of child maltreatment (2, 12, 13, 23, 24) and knowledge that single focused interventions, such as CBT, may not be effective for all children who have experienced maltreatment (97, 98).

The case studies in this research demonstrated the use of integrated interventions which engage with the child, family and professional service system. The purpose of intervention with care teams, as highlighted in case vignette 3, is to provide a space for parents and professionals to work toward a common understanding so they can better meet the needs of the child.

As a relationship-based service, Take Two recognizes that as maltreated children and young people are harmed within the context of relationships, their healing must also occur within the context of relationships. The Take Two client group has complex, multiple and chronic needs. In many cases these children are not living in safe or stable circumstances, their needs are not about protection alone, nor are they therapeutic alone; they require an approach that responds to each child with their multiple needs. This study furthers the understanding of the manner in which the NMT tools can guide the selection of interventions contributing to improved relational health with associated positive behavioral, emotional, and developmental outcomes. This study further contributes to an increased understanding of the importance of relational health and its impact on social and emotional functioning as measures by the HoNOSCA and SDQ.

### Implications for Policy and Practice

The pervasive impact of child maltreatment (12) has presented major challenges for identifying evidence informed interventions (6). The cohort involved in this cross-sectional study demonstrated severe impacts of child maltreatment. Tables 1, 2 show the comparison for baseline presentation between the Take Two cohort and national and international populations. It is clear the cohort of children in this study presented with greater symptomology, with intersectionality of family violence, disability and intergenerational trauma, and impacts of displacement.

This study provides evidence that a relational health intervention is associated with a positive change in the child's presentation across the three outcome measures utilized. Addressing the impact of the trauma of child maltreatment requires interventions which integrate attention to the individual child's presentation and to the system which surrounds the child which can become a therapeutic web (17). Each case vignette demonstrates how a focus on the individual child's presentation and on key people in their environment lead to positive changes in the child's presentation, as evidenced by the change in the child's symptoms and in the outcome measures between Time I and Time 2. This study builds upon the work of Perry (17) and Ludy-Dobson and Perry (69) who have demonstrated the importance of a focus on relational health and systemic interventions. It demonstrates the importance of these elements in addressing the harm of maltreatment. The study contributes to building the evidence base of interventions which support strengthening of relational health and the process of facilitating healing from the consequences of maltreatment.

The NMT provides an analysis of the neurobiological impact on the child and their development and the level of relational health in the child's environment. This analysis assists the clinician to determine the type of intervention which is required to assist the child. The analysis provided by NMT addresses each level of the child's social ecological system environment (100) and highlights which elements need to be addressed. Thus, the NMT provides the clinician with a guide to the intervention plan and when used at review (Time 2) it demonstrates if the intervention is leading to desired changes.

The study also highlights that intervention with this population requires more than the implementation of one evidence-based intervention due to the severe symptomology, intersectionality and need for individuated interventions based on the individual needs of the child.

The study has implications in relation to Child Protection Policy. It provides further support to the view that the concept of child wellbeing in child protection needs to be conceptualized broadly (101) and ensure that understanding of risk includes the child's relational health.

It is critical for children severely impacted by maltreatment that the intersectionality of their situation be addressed in intervention (6). Take Two clinicians utilize NMT metrics as a key part of assessment.

## Conclusion

It is critical for children severely impacted by maltreatment that the intersectionality of their situation be addressed in intervention (6).

This study illustrates the value of neurodevelopmental trauma-informed interventions in positively impacting on the relational health and current functioning of maltreated children and the potential to reduce the lifelong impact of maltreatment.

The developmentally and trauma informed intervention approach utilized by Take Two requires attention to all aspects of the child's life and the NMT model provides a framework for assessing these elements.

## Data Availability Statement

The raw data supporting the conclusions of this article will be made available by the authors, without undue reservation.

## Ethics Statement

The studies involving human participants were reviewed and approved by La Trobe University Human Research Ethics Committee- HEC 04-131. Written informed consent to participate in this study was provided by the participants' legal guardian/next of kin.

## Author Contributions

All authors listed have made a substantial, direct and intellectual contribution to the work, and approved it for publication.

## Conflict of Interest

The authors declare that the research was conducted in the absence of any commercial or financial relationships that could be construed as a potential conflict of interest. The handling editor declared a past collaboration with several of the authors AC, MF, and CR.

## Publisher's Note

All claims expressed in this article are solely those of the authors and do not necessarily represent those of their affiliated organizations, or those of the publisher, the editors and the reviewers. Any product that may be evaluated in this article, or claim that may be made by its manufacturer, is not guaranteed or endorsed by the publisher.
