# Supplementary material for: Australian Maltreated Infants and Young Children Can Achieve Positive Relational Health With Neurodevelopmentally- and Trauma-Informed Interventions Provided Within Relationally-Positive and Stable Environments
Source: Front Psychiatry. 2021 Jul 28;12:680343. doi: 10.3389/fpsyt.2021.680343 (PMC8355362; doi:10.3389/fpsyt.2021.680343)
Supplement: Supplementary file 1 [file Data_Sheet_1.docx]

**Supplementary Material - Case Study Data Collection Tool**

| UR no |  |
| --- | --- |
| Gender |  |
| ATSI status |  |
| DOB |  |
| Episode number & acceptance date |  |
| Reason for referral |  |
| Presenting Problems |  |
| Statutory order |  |
| Length of episode |  |
| Period between time 1 & 2 data |  |
| Age at time 1 |  |
| Age at time 2 |  |
| Placement at time 1 |  |
| Placement at time 2 |  |
| Changes to placement in period of episode |  |
| Other significant changes during episode |  |
| Reason for closure |  |
| NMT date, scoring clinician, rh & functional domains at time 1 |  |
| NMT date, scoring clinician, & rh & functional domains at time 2 |  |
| HoNOSCA date time 1 |  |
| HoNOSCA date time 2 |  |
| SDQ date time 1 |  |
| SDQ date time 2 |  |
| Other clinical measures |  |
| Recommended intervention & Goals |  |
| (Individual, Dyadic, Family, child-focussed carer/parent work/systems) |  |
| Fidelity to the recommended intervention |  |
| Dosage – number of sessions, frequency, time period |  |
| Systems work dosage |  |
| Diagnosis at assessment |  |
| Diagnosis at closure |  |
| Clinician name and are they still receiving service? |  |
| Other |  |
| Are there other episodes of care? |  |
